# Newer Diagnostic and Cost-Effective Ways to Identify Asymptomatic Atrial Fibrillation for the Prevention of Stroke

**DOI:** 10.7759/cureus.12437

**Published:** 2021-01-02

**Authors:** Urvish K Patel, Preeti Malik, Nidhi Patel, Priyadarshee Patel, Neev Mehta, Eseosa Urhoghide, Surya Aedma, Raja Chandra Chakinala, Shamik Shah, Kogulavadanan Arumaithurai

**Affiliations:** 1 Neurology and Public Health, Icahn School of Medicine at Mount Sinai, New York, USA; 2 Public Health, Icahn School of Medicine at Mount Sinai, New York, USA; 3 Neurology, Massachusetts General Hospital, Andover, USA; 4 Medicine, Drexel University College of Medicine, Philadelphia, USA; 5 Neurology, Drexel University College of Medicine, Philadelphia, USA; 6 Epidemiology and Biostatistics, Boston University School of Public Health, Boston, USA; 7 Internal Medicine, Hudson Regional Hospital, Secaucus, USA; 8 Internal Medicine, Carle Foundation Hospital, Urbana, USA; 9 Medicine, Geisinger Commonwealth School of Medicine, Danville, USA; 10 Medicine, Guthrie Robert Packer Hospital, Sayre, USA; 11 Neurology, Stormont Vail Health, Topeka, USA; 12 Neurology, Mayo Clinic Health System, Albert Lea, USA

**Keywords:** atrial fibrillation, zio patch, loop recording device, stroke, holter monitoring, cardiac arrhythmia, cardiac rhythm monitoring, ambulatory electrocardiography devices, electrocardiography, acute ischemic stroke

## Abstract

Atrial fibrillation (Afib) is the most common and underestimated cardiac arrhythmia with a lifetime risk of >35% after the age of 55 years and the risk continues to rise exponentially. Afib leads to stasis of blood within the atria allowing clot formation and increasing the risk for systemic embolization leading to strokes. Outcomes due to Afib can improve significantly with appropriate treatment. Thus, the need for convenient, well‐tolerated, cost‐effective cardiac monitoring for Afib is needed. The study aims to evaluate the various newer devices and compare them with traditional Holter monitoring, keeping diagnostic yield, cost-effectiveness, and patients' convenience in mind. Though Holter monitoring is simple and non-expensive, it has major limitations including limited recording capacity, inability for real-time recordings, and inconvenience to patients. Zio Patch (iRhythm Technologies, Inc; San Francisco, CA) and other loop recording devices are patient-friendly, inexpensive, and can offer real-time data for longer days. More prospective studies are needed to evaluate the sensitivity, specificity, and the actual number of patients getting benefits from newer devices by diagnosing Afib sooner and start early prevention therapy.

## Introduction and background

Atrial fibrillation (Afib) is the most common sustained cardiac arrhythmia, with a lifetime risk of 37% occurrence after the age of 55 years [1]. The causal relation between atrial fibrillation and stroke has been known for many years. Uncoordinated and rapid myocyte activity due to hyperactive electric stimulation from the SA node results in impaired contraction of the atria. This impairment leads to stasis of blood within the atria that allows clot formation to occur, thus increasing the risk for systemic embolization leading to cardio-embolic strokes [2]. 

Afib has been a major burden on the healthcare system. Though incidence throughout the general population has been relatively stable over time, the prevalence of Afib continues to rise exponentially [3]. Some of the increasing prevalence may be attributable to a modest improvement in Afib-related survival (e.g., three-year mortality rate reduction from 45% in 1993 to 42% in 2005), which is related to earlier detection and treatment of underlying conditions such as hypertension, coronary artery disease (CAD), and heart failure (HF) [3]. Afib accounts for 3-4% of all emergency department visits, with typical symptoms like syncope, palpitations, dizziness attributable to arrhythmias, etc. [4]. There are half a million hospitalizations annually in the United States for which Afib is the primary diagnosis. Afib is estimated to contribute to >100,000 deaths per year in the United States. Outpatient cardiac rhythm monitoring is an integral part of the early diagnosis and management of Afib, which is the priority in successful secondary stroke prevention [5]. This stresses the use of ambulatory cardiac monitoring devices for earlier detection of Afib.

The true epidemiological profile of Afib is incomplete and underestimated because a substantial proportion of Afib patients can be asymptomatic or without clinical manifestations (“clinically silent or subclinical Afib”) [6]. Clinically silent Afib diagnosed often during routine checkups, leaves difficult decisions for physicians on how to treat and to what extent. Currently, researchers have been focused on clinically silent and asymptomatic Afib which has shown equal outcomes regarding stroke and death. In the most recent data reported by EurObservational Research Programme (EORP)-Atrial Fibrillation Pilot General Registry, mortality at one year was more than twofold higher in asymptomatic patients than their symptomatic counterparts and was associated independently with older age and comorbidities [7].

Additionally, undiagnosed Afib can have immense complications and morbidity. Outcomes due to Afib can improve significantly with appropriate treatment, including anticoagulation to prevent systemic embolization and stroke, rhythm, and rate control for the restoration of normal rhythm. Despite guidelines from multiple societies, there is a significant gap in the care of Afib. Earlier and improved methods of detection can allow earlier initiation of appropriate therapies to prevent adverse health outcomes. Furthermore, monitored individuals, compared with non-monitored controls, had higher rates of Afib diagnosis, greater initiation of anticoagulants, but also increased health care resource utilization at one year. Better detection of paroxysmal atrial fibrillation (PAF) by prolonged cardiac monitoring can be expected to improve secondary prevention through optimized secondary preventive regimens, like oral anticoagulation for stroke patients [8]. The improved cost-effectiveness is attributable to the fact that these newly detected patients benefit from anticoagulation therapy to prevent stroke recurrence which in turn saves future costs and reduces the impairment of quality of life.

The need for convenient, well‐tolerated, cost‐effective cardiac monitoring for Afib is likely to increase as Afib becomes more prevalent [4]. Traditionally monitoring of arrhythmias has been done by a continuous electrocardiogram (ECG) monitor known as a Holter monitor, which is given to patients with a recent history of acute coronary syndrome and daily symptoms such as syncope, dizziness, and palpitations. Continuous ECG monitoring records data such as average heart rate, RR interval, and ST-segment changes for a period of 24-48 hours. Alternatively, in recent years there have been many technological advancements in this field of study. For example, we now have single and double lead ECG that can be placed directly on the chest, smartwatches, and handled devices that pair directly with your phone. These devices have automated algorithms within their specifically designed optical sensors to detect irregularities in pulse to notify the user in real-time of possible atrial fibrillations without the use of electrodes or wires. However, due to its very limited data and accuracy, it still remains unknown when this technology may be used as a primary diagnostic tool in medicine. Lastly, non-invasive continuous monitoring patch such as the Zio Patch (iRhythm Technologies, Inc; San Francisco, CA) is a single-lead ECG monitor that provides up to 14 days of continuous ECG data from a single vector. These devices are single-use, water-resistant, and allow for long term cardiac monitoring. Although this may be a promising technology, larger studies will be required to determine the efficacy of these devices in detecting arrhythmias [9].

Hence, the literature review aims to evaluate the effectiveness of various ambulatory devices in detecting asymptomatic Afib to prevent stroke.

## Review

Types of ambulatory devices

Atrial fibrillation is the most underestimated cardiac arrhythmia with a lifetime risk of >35% after the age of 55 years and the causal relationship between atrial fibrillation and stroke has been known for many years [10]. In addition to atrial fibrillation, arrhythmias such as sinus tachycardia, premature ventricular contractions, and ventricular tachycardia can all cause palpitations. Usually benign, palpitations can be a manifestation of potentially life-threatening conditions, especially if associated with dizziness, near-syncope, or syncope. Therefore, ambulatory electrocardiogram monitoring is an invaluable tool to assess and establish the diagnosis of a patient’s symptoms. There are a variety of possible ambulatory monitors to choose from such as Holter monitors, implantable loop recorders, and external loop recorders. Table 1 showed details on various ambulatory electrocardiography devices and their applications.

**Table 1:** Types of ambulatory electrocardiography device and their applications.

Types of Device	Description	Usefulness	Limitations
Holter Monitor	For daily or near-daily frequency of symptoms. Has a recording time of 24 hours, 48 hours, or one week. Available in primary and secondary care.	Suitable for patients with frequent symptoms. Less expensive. Noninvasive and no action needed from patients.	Limited recording capacity.
External Loop Recorders	For weekly frequency of symptoms. Has recording time up to 4 weeks. ECG data can be transmitted continuously over wireless networks to a remote monitoring system for evaluation. Patients need to activate by themselves during the onset of symptoms. Available only in specialized cardiac centers.	Higher likelihood of detecting arrhythmias due to prolonged monitoring (in comparison to Holter). Noninvasive.	Not suitable for conditions like syncope when patients cannot activate the device.
Implantable Loop Recorders	For rare (monthly) frequency of symptoms. Has the longest recording time for up to 3 years. Available only in specialized cardiac centers.	Minimally invasive. Both automatic and patient activated methods are supported.	Most expensive among the three device types.
Zio Patch	A single-lead ECG monitor that has no external leads or wires. The patch is stuck on the patient’s left pectoral region and can record a continuous beat-to-beat ECG, making it useful for monitoring cardiac rhythm, for up to fourteen days.	Zio Patch has a higher diagnostic yield to detect Afib and prevention of strokes per year. This would result in significant yearly savings in direct medical costs.	The data of Zio Patch is analyzed offline after the completion of the monitoring.

Holter Monitor

Traditionally, monitoring of arrhythmias has been done by a continuous ECG monitor known as a Holter monitor. The most common monitors allow for continuous registration of three or more leads for 24-48 hours while newer monitors allow for continuous ECG monitoring for up to two weeks [11]. A benefit in extending the time of ECG registration helps improve the diagnostic yield of Holter monitoring, especially for infrequent but recurrent rhythm disturbances [12]. The Holter monitor aids in the detection of arrhythmias and ST-segment changes help to assess the therapeutic efficiency of antiarrhythmic agents and helps to evaluate pacemaker malfunctions [13]. A primary advantage of using Holter monitoring is that it aids in quantifying the real burden of arrhythmia and could help the clinician in making therapeutic decisions for disabling arrhythmias that occur frequently [14]. However, despite this advantage, some limitations include the relatively brief duration of monitoring, limited recording capacity, and inability to transmit real-time data to the attending cardiac unit, and the need for close collaboration between the patient and the healthcare professional [14]. Additionally, these may cause physical discomfort for patients due to the large size of the monitor and electrodes that need to be taped to various areas on the skin that may irritate.

Loop Recorders

Loop recorders are event recorders that work by continually analyzing the ECG and retaining information pertinent to relevant arrhythmias. This is possible through predefined algorithms and registration of the ECG a few minutes prior to the onset of the arrhythmia [14]. These recorders can be activated by the patient when he/she experiences the symptoms and can therefore reliably document a correlation between symptoms and arrhythmia.

Internal Loop Recorders

Implantable loop recorders is a subcutaneous monitoring device used to monitor electrical activity of the heart over an extended period of time, compared to the fixed picture of electrical activity seen with ECGs [15]. These devices can record for up to three years. An implantable loop recorder can store patient-activated episodes, automatically activated episodes or a combination of the two. Some benefits of an implantable loop recorder are that it does not need to be removed during certain activities such as showering or swimming and it can help identify significant cardiac rhythm abnormalities when the patient is sleeping [15]. Additionally, unlike the Holter monitor, an implantable loop recorder has a higher likelihood of detecting arrhythmias due to prolonged monitoring and the ability to detect atrial fibrillation recurrences, as they can be silent and unpredictable [16]. However, it may be affected by false episode detection due to artifacts and they only allow the registration of one lead, rendering the interpretation of the ECG difficult in some cases [14]. Likewise, unlike the Holter monitor, it is far more efficient and reliable at identifying abnormal rhythms [15] and data transmission to a distant diagnostic station is simple [14]. This device may be useful for noncompliant patients, as there are no external parts to be worn [17].

External Loop Recorders

External loop recorders can be connected to a belt around the chest, without the need for traditional electrodes, and can monitor the ECG for a maximum of 30 days [18]. Additionally, because this device relies on the patient activating it, it is not suitable for syncope or other conditions in which the patient is unable to activate the device. An advantage of using this device is that ECG data can be transmitted continuously over wireless networks to a remote monitoring system for evaluation.

Zio Patch

The Zio Patch is a single-lead ECG monitor that has no external leads or wires. The patch adheres to the patient’s left pectoral region and can record a continuous beat-to-beat ECG, making it useful for monitoring cardiac rhythm, for up to fourteen days. Similar, to the Holter monitor, the data from the Zio Patch is analyzed offline after the completion of the monitoring. However, the Zio Patch has a higher diagnostic yield than the Holter monitor [19]. The patch provides a high diagnostic yield for arrhythmia because the diagnostic yield of continuous loop-recording decreases rapidly after two weeks of monitoring and monitoring beyond seven days provides only an additional 3.9% of patients with a diagnosis [20]. In an epidemiologic study done on the older general population, it was found that atrial fibrillation was detected in 4% of those with no prior history, and 38% of newly detected atrial fibrillation was first found on days three-14 of monitoring with the Zio Patch [21]. Additionally, a single monitoring episode of 12 days was adequate for estimating the extent of supraventricular and ventricular ectopy [21]. Table 2 described the studies measuring the diagnostic yield of ambulatory ECG monitoring by utilizing newer devices.

**Table 2:** Studies measuring diagnostic yield of ambulatory ECG monitoring. VT: ventricular tachycardia, PAF: paroxysmal atrial fibrillation, SVT: supraventricular tachycardia, Afib: atrial fibrillation, APB: atrial premature beat, TTE: transthoracic echocardiography, ECG: electrocardiogram, AD: absolute difference, OR: odds ratio, IQR: interquartile range

Author, Year, and Country	Sample Size	Study Type and Duration	Aim/Objective	Outcomes	Results
Schreiber et al., 2013 (USA) [4]	174	Multicenter Prospective Observational study; February 2011-February 2012	To determine the diagnostic yield of Zio Patch and to determine the value of prolonged monitoring of low-risk discharged ED patients with possible cardiac arrhythmia.	Significant arrhythmias as ventricular tachycardia (VT) ≥4 beats, paroxysmal atrial fibrillation (PAF), supraventricular tachycardia (SVT) ≥4 beats, ≥3-sec pause, 2nd-degree Mobitz II or 3rd degree AV block, or symptomatic bradycardia. Serious arrhythmias were defined as VT >120 for 30 seconds, Complete or 3rd-degree heart block, symptomatic second-degree heart block, type II, pause >6 seconds, and symptomatic bradycardia <40 beats per minute for >30 seconds.	The average age 52.2 (± 21.0) years and 55% were female. The most common indications for device placement were palpitations (44.8%), syncope (24.1%), and dizziness (6.3%). 47.7% had ≥1 arrhythmia and 9.8% were symptomatic at the time of their arrhythmia. 5.2% had ≥2 arrhythmias. 7 patients required immediate physician notification for serious arrhythmias. 93 (53.4%) of symptomatic patients did not have any arrhythmia during their triggered events. The overall diagnostic yield was 63.2%.
Gladstone et al., 2015 (USA) [22]	237	Multicenter randomized controlled trial.	To predict which cryptogenic stroke or TIA patients have the highest probability of subclinical Afib. Data from the EMBRACE trial was used to investigate the association between Holter-detected atrial ectopic activity, on the subsequent detection of Afib by 30-day ECG monitoring, and during clinical follow-up at 90 days and 2 years.	Primary: Detection of ≥1 episode of atrial fibrillation or flutter lasting ≥30 s by 30-day ECG monitoring or clinically within 90 days post-randomization. Secondary: Afib ≥30 s detected by 30-day ECG, Afib ≥2.5 minutes detected by 30-day ECG, and Afib detected by any means within 2 years of clinical follow-up.	Primary: Median baseline APB count was 66 (IQR, 18-309) in the entire cohort, higher in patients who were subsequently found to have Afib (629 beats/24 h {IQR, 142-1973}) compared with those without Afib (45 beats/24 h {IQR, 14-250}); p<0.001. Secondary: Afib ≥30 s on the 30-day ECG monitor alone (p<0.0001) and the more robust outcome of Afib ≥2.5 minutes on 30-day ECG (p=0.0005), and for Afib detection by any means at 2 years (p=0.0027). Overall, the 90-day Afib detection rate in the intervention group was 16% but it was highly dependent on the baseline APB count: the predicted probability of Afib was 7% to 9% in patients with <100 APBs/24 h, 9% to 24% in those with 100 to 499 APBs/24 h, 25% to 37% in those with 500 to 999 APBs/24 h, 37% to 40% in those with 1000 to 1499 APBs/24 h, and it reached a plateau ≈40% in those with ≥1500 APBs/24 h.
Kaura et al., 2019 (UK) [23]	Patch-based monitoring group:56. Holter monitoring group:60	Open-label randomized controlled trial February 2016-February 2017 for 90 days	Compare 14-day ECG monitoring patch (Zio Patch) with short-duration Holter monitoring for the detection of PAF.	Primary: Detection of one or more episodes of ECG-documented PAF lasting at least 30 s within 90 days in each of the study arms. Secondary: PAF lasting at least 30 s within 28 days in each of the study arms and PAF lasting at least 30 s detected on the patch-based monitoring or short-duration Holter monitor within 90 days in patients who underwent both ECG monitoring strategies and A budget impact analysis from the healthcare perspective was performed	Primary: The rate of detection of PAF at 90 days was 16.3% in the patch-based monitoring group (seven patients) compared to 2.1% in the short-duration Holter monitoring group (1 patient), with an odds ratio of 8.9 (95% CI: 1.1-76.0; p=0.026) Secondary: An economic model demonstrated that implementation of the Zio Patch service would result in 10.8 more strokes avoided per year compared to current practice with Holter monitoring with an associated yearly saving in direct medical costs of £113,630, increasing to £162,491 over 5 years.
Kamel et al., 2013 (USA) [24]	40	Pilot randomized controlled trial. October 29, 2009-May 24, 201; 21 days with follow up at 3 months and 1 year	To establish the safety and feasibility of Cardionet Mobile Cardiac Outpatient Telemetry 20 patients wear the for 21 days and 20 patients to get routine care.	Primary: primary feasibility outcomes were enrollment of 40 patients in 2 years, completion of assigned monitoring in ≥70% of patients, and full follow-up for ≥90% of patients. The primary safety outcome was any adverse event resulting directly from the use of the cardiac monitoring device. Secondary: New diagnoses of Afib within 3 months and 1 year	64% Overall compliance. No patient diagnosed with Afib, 2 patients had brief episodes (<10 seconds) of atrial tachycardia, and 2 patients had non-sustained ventricular tachycardia. No serious adverse event occurred that was attributable to the monitoring intervention.
Baturova et al., 2016 (USA) [25]	110	Post-hoc analysis from a previous prospective case-control study.	Investigate clinical, ECG, and TTE characteristics associated with paroxysmal Afib in ischemic stroke patients.	To assess predictors of paroxysmal atrial fibrillation using non-invasive surface ECG and transthoracic echocardiography to select candidates for atrial fibrillation screening	Primary: Atrial fibrillation history was independently associated with vascular diseases (OR: 4.10; 95% CI: 1.32-12.78; p=0.015), P wave terminal force in lead V1 > 40 mm*ms (OR: 4.04; 95% CI: 1.34-12.14; p=0.013) and left atrial volume index (OR: 1.08; 95% CI: 1.03-1.13; p=0.002). Left atrial volume index remained an independent predictor of atrial fibrillation detected after stroke (OR: 1.09; 95% CI: 1.02-1.16; p=0.017).
Halcox et al., 2017(UK)[26]	1001 patiensiECG:500 Routine care: 501	Randomized controlled trial for 12 months	To evaluate the efficacy of AliveCor Kardia device (a smartphone/tablet-based single-lead electrocardiographic capture system) vs routine clinical care (RC) in patients >65 years of age with ≥1 additional stroke risk factor.	Primary: The primary endpoint was time to diagnosis of Afib. Afib was defined as a 30-second iECG recording with irregular rhythm without p waves Secondary: Incidence of Adverse vascular events which were either reported at the time of event or were identified by telephone at 12, 32, and 52 weeks, with confirmation from source clinical records.	Primary: 74% overall compliance. 19 patients in the iECG group were diagnosed with Afib over the 12-month study period vs 5 in the RC arm (HR: 3.9; 95% CI: 1.4-10.4; p=0.007) at a cost per Afib diagnosis of $10 780 (£8255). Secondary: There was a similar number of stroke/transient ischemic attack/systemic embolic events (6 vs 10, iECG vs RC; HR:0.61; 95% CI: 0.22-1.69; p=0.34).
Reed et al., 2018 (Scotland) [27]	86	Prospective pilot study. November 17, 2015-June 16, 2017	This study investigates diagnostic yield, event prevalence, patient satisfaction and compliance, and influence on resource utilization of an ambulatory patch monitor (Zio XT monitor).	The primary endpoint was symptomatic significant arrhythmia at 90-day follow-up.	90-day diagnostic yield for symptomatic significant arrhythmia was 10.5% (95% CI: 4.0-16.9; 9 of 86) vs 2.0% (95% CI: 0.9-3.1; 12 of 603) in the comparator group. 24 patients (27.9%) had a significant arrhythmia (five serious); 26 patients (30.2%) had serious outcomes (major adverse cardiac event and/or death). The patch would significantly reduce requirements for standard outpatient ambulatory ECG monitoring. 56 of 76 returned patches had a diagnostic finding within ± 45s of a triggered/diary event (73.7% diagnostic utility; 95% CI: 63.7-83.6); 34 of 56 (61%) for sinus rhythm or ectopic beats only
Pradhan et. al, 2019 (USA) [28]	363	Single-center Retrospective study. October 2014 to February 2016	To describe the duration of ZIO XT Patch use by age and to compare its time to arrhythmia detection with the Holter monitor in a pediatric population.	Demographics, as well as diagnostic data including duration of ZIO use, time to the first arrhythmia, and arrhythmias detected. SVT was defined as 3 or more consecutive ectopic beats arising from proximal to the bundle of His. VT was defined as 3 or more consecutive ectopic beats arising distal to the bundle of His.	The median age was significantly different between the ZIO (12.7 years) and Holter (4.9 years) within 72 hours (n=15). The majority of arrhythmias (57%) detected by ZIO were after 24 h (p<0.0001). All arrhythmias detected by the Holter monitor occurred within 24 h (p<0.0001).
Turakhia et. al, 2013 (USA) [29]	26,751	Cross-sectional study. January 1, 2011-December 31, 2011	To evaluate compliance, analyzable signal time, the interval to arrhythmia detection, and diagnostic yield of the Zio Patch.	Arrhythmia first occurrence, first symptomatic occurrence (if occurring 45 seconds before or after patient triggering), and longest duration. The total wear time was calculated from the point of activation to the point of the last recorded analyzable signal.	The mean wear time was 7.6±3.6 days, and the median analyzable time was 99% of the total wear time. Arrhythmias were detected in 60.3% of patients, 46% had single arrhythmia, 11.5% had multiple. 7.5% had chronic Afib. 29.9% had their first arrhythmia and 51.1% had their first symptom-triggered arrhythmia after the initial 48-hour period. Compared with the first 48 hours of monitoring, the overall diagnostic yield for Zio Patch was greater for any arrhythmia (62.2% vs 43.9%; p<0.0001) and for any symptomatic arrhythmia (9.7% vs 4.4%; p<0.0001).
Steinhubl et. al, 2018 (USA) [30]	2659	Randomized clinical trial and prospective matched observational cohort study. November 17, 2015- October 4, 2016	To determine the effect of a self-applied wearable ECG patch in detecting Afib and the clinical consequences associated with such a detection strategy.	Primary: Incidence of a new diagnosis of Afib at 4 months among those randomized to immediate monitoring vs delayed monitoring. Secondary: New Afib diagnosis at 1 year in the combined actively monitored groups vs matched observational controls. Other: New prescriptions for anticoagulants and health care utilization (outpatient cardiology visits, primary care visits, or Afib-related emergency department visits and hospitalizations) at 1 year.	New Afib was identified by 4 months in 3.9% of the immediate group vs 0.9% in the delayed group (AD: 3.0%; 95% CI: 1.8-4.1%). At 1-year, new Afib diagnosed in 109 monitored and 81 unmonitored. Active monitoring was associated with increased initiation of anticoagulants (AD: 2.0; 95% CI: 1.9-2.2), outpatient cardiology visits (AD: 7.5; 95% CI: 7.2-7.9), and primary care visits (AD: 0.9; 95% CI: 0.4-1.5). There was no difference in Afib-related ED visits and hospitalizations.
Rosenberg et. al, 2013 (USA) [9]	74	Single-center Prospective study. April 27, 2011-May 25, 2012	To determine if Zio Patch would be well tolerated and function as well as a Holter monitor in the first 24 hours of use in terms of the detection of Afib and other arrhythmias. To determine if additional days of monitoring would be tolerated and yield meaningful clinical findings.	Significant arrhythmias were defined as Afib or atrial flutter, other supraventricular tachycardias (not including Afib or atrial flutter) for >4 beats, sustained ventricular tachycardia (>4 beats), junctional rhythm, sinus bradycardia (<50 beats/min), and complete or high-grade heart block.	The Zio Patch was well tolerated, with a mean monitoring period of 10.8 ± 2.8 days. During the first 24 hours period, there was a significant difference in the mean Afib burden estimated by the Zio Patch and the Holter monitor (p<0.0001). Afib events were identified in 18 additional individuals, and the documented pattern of Afib (persistent or paroxysmal) changed in 21 patients after Zio Patch monitoring. As a result of the findings from the Zio Patch, 28.4% of patients had a change in their clinical management.
Kaura et. al, 2019 (UK) [31]	120	Open-label randomized controlled trial. February 2016-February 2017	To comparing a 14-day Zio Patch, with Holter monitoring for the detection of PAF.	The primary outcome was the detection of one or more episodes of ECG-documented PAF lasting at least 30 s within 90 days in each of the study arms.	The rate of detection of PAF at 90 days was 16.3% in Zio Patch compared to 2.1% in the short-duration Holter monitoring group (OR: 8.9; 95% CI: 1.1-76.0; p=0.026). Zio Patch service would result in 10.8 more strokes avoided per year compared to current practice with Holter monitoring with an associated yearly saving in direct medical costs of £113,630, increasing to £162,491 over 5 years.

Cost-effectiveness

Early detection and timely treatment of arrhythmias are important to reduce the burden of cardiac disease and lower healthcare costs. ECG monitoring beyond the 24-48-hours Holter monitor can improve the detection of arrhythmias, however, prolonged monitoring beyond eight to 14 days is generally not cost-effective [4,9,22-31]. Therefore, wearable patch monitors such as the Zio Patch, that record at least eight days of ECG data are cost-effective alternatives to the traditional Holter monitors and loop recorders.

In an economic model derived from a randomized controlled study comparing the efficacy of a Holter monitor to the Zio Patch for the detection of PAF after a transient ischemic attack/ischemic stroke, it was found that the implementation of the Zio Patch would prevent 10.8 more strokes per year when compared to the current practice with Holter monitoring [23]. This would result in a yearly saving in direct medical costs of £113,630 ($146,963), increasing to £162,491 ($210,157) over five years [23]. Additionally, Brignole et al. noted that while the 24-48 hours Holter monitor has a relatively low set-up cost, it is expensive in terms of cost per diagnosis [32]. Arnold et al. also state that although it is fairly common to repeat Holter monitoring after the first Holter procedure due to inconclusive results, repeat monitoring did not yield a diagnosis, and patients continued to experience clinical events that led to substantial health costs [33]. In another study comparing the efficacy of the Holter monitor to a 14-day adhesive patch, it was found that the adhesive patch monitor detected 96 arrhythmia events compared with 61 arrhythmia events by the Holter monitor (p<0.001) [34]. This emphasizes the importance of effective diagnosis and treatment in reducing healthcare costs, morbidity, and mortality associated with cardiac arrhythmias. Additionally, the study comparing seven-day-Holter monitoring (7-d-Holter) to a standard 24-hour-Holter to detect PAF, it was found that the seven-day-Holter in patients with cerebral ischemia is cost-effective [8]. The cost-effectiveness is due to the increased detection which leads to the implementation of improved antithrombotic regimes that work to avoid recurrent strokes and decrease quality of life impairment. This also emphasizes the role that a seven to 14-day monitoring modality, such as the Zio Patch, can play in reducing healthcare costs. 

In addition to the increased healthcare costs associated with the use of the Holter monitor, studies have found that there is an increased cost in monitoring periods beyond two weeks. For instance, the costs can range up to $5832 per new diagnosis versus a $98 cost per patient diagnosis over an initial seven days and $576 over a 14-day period [34]. Therefore, the Zio Patch is likely to achieve a reasonable diagnostic yield compared to loop recorders which typically monitor from up to a month to three years.

## Conclusions

Our knowledge of the true (“clinically silent or subclinical Afib”) prevalence of Afib is underestimated and represents the tip of the iceberg. Holter monitoring is helpful yet limited by duration, recording capacity, and inability to transmit real-time data. External loop recorder and Zio Patch are better alternatives due to convenient and accurate recording and portability. These newer devices would result in more stroke prevention per year and are more cost-effective in comparison with Holter monitoring. Although these newer devices are proven effective to identify Afib, more prospective studies should be planned to evaluate sensitivity, specificity, and the role of these devices to begin early management in the direction of stroke prevention, and patients’ satisfaction.
